# High-Level Quinolone-Resistant *Haemophilus haemolyticus* in Pediatric Patient with No History of Quinolone Exposure

**DOI:** 10.3201/eid2801.210248

**Published:** 2022-01

**Authors:** Emi Tanaka, Yuji Hirai, Takeaki Wajima, Yu Ishida, Yoshiaki Kawamura, Hidemasa Nakaminami

**Affiliations:** Tokyo University of Pharmacy and Life Sciences, Tokyo, Japan (E. Tanaka, T. Wajima, H. Nakaminami);; Meijo University, Nagoya, Japan (E. Tanaka, T. Wajima);; Tokyo Medical University Hachioji Medical Centre, Tokyo (Y. Hirai, Y. Ishida);; Aichi Gakuin University, Nagoya, Japan (Y. Kawamura)

**Keywords:** antimicrobial resistance, quinolone resistance, bacteria, *Haemophilus* spp., *Haemophilus haemolyticus*, *Haemophilus influenzae*, quinolone, respiratory infections

## Abstract

The prevalence of antimicrobial resistance among *Haemophilus* spp. is a critical concern, but high-level quinolone-resistant strains had not been isolated from children. We isolated high-level quinolone-resistant *H. haemolyticus* from the suction sputum of a 9-year-old patient. The patient had received home medical care with mechanical ventilation for 2 years and had not been exposed to any quinolones for >3 years. The *H. haemolyticus* strain we isolated, 2019-19, shared biochemical features with *H. influenzae*. However, whole-genome analysis found this strain was closer to *H. haemolyticus.* Phylogenetic and mass spectrometry analyses indicated that strain 2019-19 was in the same cluster as *H. haemolyticus.* Comparison of quinolone resistance–determining regions showed strain 2019-19 possessed various amino acid substitutions, including those associated with quinolone resistance. This report highlights the existence of high-level quinolone-resistant *Haemophilus* species that have been isolated from both adults and children.

The genus *Haemophilus* includes 9 bacterial species that cause infections only among humans: *H. influenzae*, *H. aegyptius*, *H. haemolyticus*, *H. parainfluenzae*, *H. parahaemolyticus*, *H. paraphrohaemolyticus*, *H. pittmaniae*, *H. sputorum*, and *H. ducreyi* (*1*). Among them, *H. influenzae* is a notable causative pathogen of respiratory infections and otitis media in children (*2*–*4*). *H. haemolyticus*, which is genetically similar to *H. influenzae* and coexists with *H. influenzae* in the upper respiratory tract (*1*,*5*), is considered a commensal bacterium, and its pathogenicity has not been widely examined. However, some previous studies have reported that *H. haemolyticus* can be misidentified as *H. influenzae* in the clinical setting (*6*–*8*).

β-lactams and quinolones are commonly used to treat infections caused by both *H. influenzae* and *H. haemolyticus*. Recently, an increase in *H. influenzae* strains with reduced susceptibility to quinolones has been reported (*9*–*11*). Moreover, high-level resistant strains (MIC for levofloxacin ≥8 μg/mL) of *H. influenzae* have also emerged (*12*–*15*) but have been isolated only from adult case-patients (*11*,*12*). Recent nationwide surveillance in Japan indicated that quinolone-resistant *H. influenzae* had not been isolated among pediatric patients (*16*). Although low-susceptibility strains of *H. haemolyticus* have emerged, a high-level resistance strain had not been isolated from a pediatric patient (*17*). We isolated the *H. haemolyticus* strain 2019-19, which showed high-level resistance to quinolones, from a pediatric patient in an acute care hospital in Tokyo, Japan, and analyzed the features of the strain and case background of the patient.

## Materials and Methods

### Patient Characteristics

A 9-year-old girl with severe motor and intellectual disabilities, hypothyroidism, and chronic respiratory disease was hospitalized for hypoxic ischemic encephalopathy in the Tokyo University of Hachioji Medical Centre (Tokyo, Japan). The patient had been under mechanical ventilation related to tracheostomy since she was 7 years of age and had not been administered any quinolones for >3 years before hospitalization. Because the patient had a fever with increased sputum production after 3 days of hospitalization, we obtained a suction sputum culture and administered ampicillin/sulbactam intravenously to her for 1 week. The patient was discharged because her fever resolved after 5 days of hospitalization.

### Bacterial Isolation and Culture Conditions

We isolated *H. haemolyticus* (strain identification 2019-19) from the suction sputum and identified it as quinolone-resistant *H. influenzae* by routine laboratory testing using a MicroScan WalkAway system (Siemens, https://www.siemens.com). Because quinolone-resistant *H. influenzae* had never been isolated from a pediatric patient, we performed a detailed susceptibility test for 2019-19 by the broth microdilution method. For controls in the biochemical test, we used *H. influenzae* GTC 14202^T^ (DSM 4690^T^) and *H. haemolyticus* GTC 15009^T^ (NCTC 10659^T^) type strains purchased from Gifu University (https://www.gifu-u.ac.jp). In addition, we used *H. influenzae* ATCC 49247 and Rd as quality control strains for antimicrobial susceptibility testing. We cultured the isolates overnight on chocolate agar at 37°C in a 5% CO_2_ atmosphere and stored them in 10% skim milk at −80°C until use. This study was approved by the research ethics committees at the Tokyo University of Pharmacy and Life Sciences (case no. 16-12).

### Antimicrobial Susceptibility Test

We measured MICs by broth microdilution method as described by the Clinical and Laboratory Standards Institute (*18*). As tested agents, we used ampicillin, amoxicillin, clavulanic acid, cefotaxime, meropenem, clarithromycin, azithromycin, levofloxacin, tosufloxacin, and moxifloxacin. In addition, we used PAβN (Phe-Arg β-naphthylamide dihydro-chloride; Sigma-Aldrich; https://www.sigmaaldrich.com) and reserpine (Sigma-Aldrich) as efflux pump inhibitors. We set antimicrobial-susceptible breakpoints according to Clinical and Laboratory Standard Institute criteria (*18*).

### Genomic Analysis

We extracted genomic DNA using a Wizard Genomic DNA purification kit (Promega, https://www.promega.com) and sequenced it using GridION (Oxford Nanopore Technologies, https://nanoporetech.com) and DNB Seq-G400 (MGI Tech, https://en.mgi-tech.com) according to manufacturer instructions. We assembled the sequenced data with Unicycler version 0.4.7 (https://github.com/rrwick/Unicycler) with default parameters, assessed the quality of the genome using CheckM, version 1.0.12 (https://github.com/Ecogenomics/CheckM), and annotated the assembled genome sequence using DDBJ’s DFAST Fast Annotation and Submission Tool; https://dfast.nig.ac.jp). The obtained and annotated sequence data were registered in the DDBJ database under DDBJ/EMBL/GenBank accession number AP024093.

Because the genome sequence of the type strain was not available, we used *H. haemolyticus* NCTC 10839, along with *H. influenzae* ATCC 33391^T^, to compare the entire genomes with strain 2019-19, using Easyfig version 2.2.2 (*19*). We calculated the average nucleotide identity (ANIb) algorithm using BLAST (https://blast.ncbi.nlm.nih.gov/Blast.cgi) with JSpeciesWS (*20*) and digital DNA-DNA hybridization (dDDH) using the type strain genome server (*21*). In addition, we estimated the presence of CRISPR sequences and *cas* genes in the genome using the CRISPRfinder program (*22*).

### Phylogenetic Analysis of Typical Genes

We illustrated a phylogenetic dendrogram using typical genes (16S rDNA sequence, *adk*, *pgi*, *recA*, *infB*, *gyrA*, *gyrB*, *parC*, *parE*, and *ftsI*) with Clustal Omega alignment and the neighbor-joining method of Jukes-Cantor using Geneious Prime 2019 (Biomatters, https://www.geneious.com). We selected the 16S rDNA sequence, *adk*, *pgi*, *recA*, and *infB* because they were used in a previous classification study (*1*,*23*). In addition, we used *gyrA*, *gyrB*, *parC*, *parE*, and *ftsI* as antimicrobial-targeting genes. We used nucleotide sequences of *Escherichia coli* ATCC 11775^T^ as an outgroup.

### Biochemical Test

We used an API NH kit (bioMérieux, https://www.biomerieux.com) to assay biochemical characteristics, prepare bacterial cultures, and interpret the results according to manufacturer protocols. We evaluated use of the V factor, X factor, and several nutrients using *Haemophilus* ID Quad with growth factors agar (BD Biosciences, https://www.bdbiosciences.com). We cultured the agar plates overnight at various temperatures (4°C, 16°C, 25°C, 37°C, and 42°C).

### Mass Spectrometry

We prepared samples by ethanol/formic acid extraction. One loop of bacteria was suspended in 300 μL distilled water and 900 μL of ethanol was added into the suspension. After centrifuging and discarding the supernatant, we mixed 20 μL each of 70% formic acid and acetonitrile. Next, we applied 1 μL of supernatant on the target plate and mixed it with 1 μL HCCA matrix (Bruker, https://www.bruker.com). We obtained the spectrum using a matrix-assisted laser desorption/ionization time-of-flight (MALDI-TOF) MS Bruker autoflex maX and analyzed the phylogenetic tree using MALDI Biotyper Compass Explorer version 4.1.60 (Bruker).

### Amino Acid Substitutions of GyrA, GyrB, ParC, and ParE

We estimated amino acid substitutions of DNA gyrase and topoisomerase IV from the nucleotide sequences of *gyrA, gyrB, parC*, and *parE*. We compared the substitutions with *H. haemolyticus* CCUG 12834^T^ or ATCC 33390^T^ (DDBJ/EMBJ/GenBank accession no. LYCK01000011, LYCK01000013, or JTLY01000001), NCTC 10839 (LS483458), M19346 (CP031243), and M28486 (CP031238).

## Results 

### Genomic Analysis

To further characterize the 2019-19 strain in detail, we determined the whole-genome sequence by next generation sequencing (Figure). The genome size was 1,895,310 bp, comprising 1,764 protein-coding sequences, 19 rRNAs, 57 tRNA, and a CRISPR sequence (Figure panel A). Comparing the whole-genome sequence of 2019-19 with that of *H. influenzae* ATCC 33391^T^ resulted in a dDDH score of 43.4% (95% CI 40.9%–46.0%) and ANIb score of 90.90%, suggesting an extremely low similarity. In contrast, although we observed a large inversion, we found 2019-19 comparatively strongly related to *H. haemolyticus* NCTC 10839 (Figure, panels B, C). The identity scores for 2019-19 with *H. haemolyticus* type strain ATCC 33390^T^ were 64.1% (95% CI 61.2%–66.9%) for dDDH and 95.38% for ANIb, suggesting great similarity. To further clarify the genetic classification, we performed phylogenetic analyses using typical species housekeeping genes (16S DNA sequence, *adk*, *pgi*, *recA*, *infB*) and antimicrobial targeting genes (*gyrA*, *gyrB*, *parC*, *parE*, and *ftsI*) (Appendix Figure 1). In all phylogenetic trees using these genes, 2019-19 was classified in the same cluster as *H. haemolyticus.* In addition, 2019-19 contained a CRISPR sequence but not an IgA protease, which is a putative marker for distinguishing it from *H. influenzae* (data not shown) (*8*).

**Figure:**
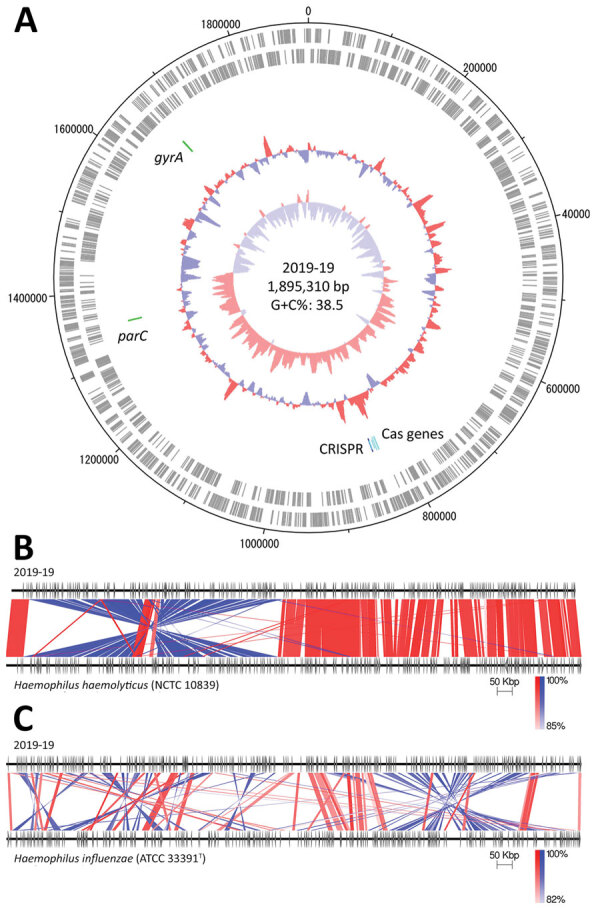
Genomic analysis of *Haemophilus haemolyticus* strain 2019-19 from a 9-year-old girl in Japan. A) Circular map of the whole-genome sequence. The outermost circle shows the number of nucleotides, the second circle shows coding sequences on the plus strand, and the third circle shows coding sequences on the minus strand. The innermost circle represents the G+C skew (%) and second innermost circle, G+C content (%); green zones show the locations of *gyrA* and *parC*, and blue and light blue zones show CRISPR-Cas–associated genes. Map drawn using Artemis DNA Plotter (Wellcome Sanger institute, Hinxton, UK). G+C, guanine + cytosine. B, C) Comparison between the whole genomes of 2019-19 and *H. haemolyticus* NCTC 10839 (B) and *H. influenzae* ATCC 33391^T^ (C), created using Easyfig version 2.2.2 (*19*). Red indicates matches in the same direction; blue indicates inverted matches; white areas indicate nonmatches.

### Biochemical Tests

Because we identified this strain as *H. influenzae* by routine laboratory testing, we also investigated the biochemical characteristics of 2019-19 (Table 1). In comparison with the type strains of both *H. haemolyticus* and *H. influenzae*, all biochemical results completely matched with *H. influenzae.* In addition, we identified species on the basis of these results using Apiweb (bioMérieux), which indicated that 2019-19 had 99.9% identity with *H. influenzae.* According to the method for *H. influenzae* described elsewhere, 2019-19 was determined to be biotype II (*1*).

**Table 1:** Antimicrobial susceptibility of *Haemophilus haemolyticus* strain 2019-19 from a 9-year-old girl in Japan

Agent	MIC, μg/mL	Decision†
Ampicillin	0.25	S
Amoxicillin/clavulanic acid	0.125	S
Cefotaxime	<0.063	S
Meropenem	<0.063	S
Clarithromycin	4	S
Azithromycin	0.5	S
Levofloxacin	16	NS
Tosufloxacin	≥64	ND
Moxifloxacin	64	NS

*S, susceptible; ND, not determined; NS, nonsusceptible †Decided by Clinical Laboratory and Standards Institute criteria (*18*).

### Mass Spectrometry

MALDI-TOF mass spectrometry analysis is one of the most reliable methods for identifying bacterial species (*24*,*25*). The bacterial protein profile was assayed to identify the bacterial species of 2019-19 using this method. In comparison with the database using MALDI Biotyper Compass Explorer version 4.1.60, 2019-19 matched with *H. haemolyticus* CCUG 12834^T^ with a score 2.21 and was identified as *H. haemolyticus*. Furthermore, in a phylogenetic dendrogram drawn with representative type strains of *Haemophilus* spp. (Appendix Figure 2), 2019-19 was located close to the *H. haemolyticus* type strain ATCC 33390^T^.

### Quinolone Resistance Mechanisms

Antimicrobial susceptibility testing of strain 2019-19 showed high MIC values (16 to ≥64 μg/mL) to levofloxacin, tosufloxacin, and moxifloxacin and susceptibility to antimicrobial agents including penicillins, cephems, and macrolides, but not to quinolones (Table 2). We investigated amino acid substitutions in quinolone-targeting proteins (GyrA, GyrB, ParC, and ParE) by comparing the sequences of *H. haemolyticus* CCUG 12834^T^ or ATCC 33390^T^, NCTC10839, M19346, and M28486 (Table 3; Appendix Figure 3). The results revealed that 2019-19 had various amino acid substitutions in GyrA, GyrB, ParC, and ParE, including amino acid substitutions (Ser84Leu, Asp88Tyr in *gyrA* and Ser84Arg in *parC*) relevant to reducing susceptibility to quinolones (*13*,*17*). We measured the MICs of quinolones in the presence of the efflux pump inhibitors reserpine and PAβN to determine whether the efflux system affected quinolone resistance. There was no substantial difference in the presence or absence of inhibitors. To investigate the origin of this strain, we compared quinolone target genes among *Haemophilus* spp.; however, we obtained no evidence of recombination (data not shown).

**Table 2:** Comparison of biochemical characteristics of *Haemophilus* *haemolyticus* strain 2019-19 from a 9-year-old girl in Japan and reference species*

Characteristics	2019-19	GTC 14202^T^	GTC 15009^T^
V-factor requirement	+	+	+
X-factor requirement	+	+	+
Indole production	+	+	-
Urease	+	+	+
Lipase	–	–	–
Ornithine decarboxylase	–	–	–
Alkaline phosphatase	+	+	+
Proline arylamidase	–	–	–
β-galactisidase	–	–	–
γ‐glutamyltransferase	–	–	–
Acid source			
D-glucose	+	+	+
D-fructose	+	+	+
Maltose	–	–	+
Sucrose	–	–	–
Growth temperature			
4°C	–	–	–
16°C	–	–	–
25°C	–	W	–
37°C	++	++	++
42°C	–	–	–
CO_2_ enhances growth	–	–	–
Hemolysis	–	–	+

*W, weak; –, negative; + positive.

**Table 3:** Amino acid substitutions in quinolone target protein of *Haemophilus haemolyticus* strain 2019-19 from a 9-year-old girl in Japan*

GyrA	GyrB	ParC	ParE
S84L	A567T	S84R	P439S
D88Y	N631S	S138T	L502F
H212Y	A725V	V214I	D596N
T251S		V270I	A599S
D740E		D442N	
S784N		M591I	
		A641E	

*Compared with *H. haemolyticus* CCUG 12834^T^ or ATCC 33390^T^, NCTC10839, M19346, and M28486

## Discussion

We analyzed high-level quinolone-resistant *H. haemolyticus* strain 2019-19 isolated from a pediatric patient in an acute care hospital in Japan. The patient had several coexisting diseases and had been under tracheotomy for 2 years but had not been exposed to quinolone for the previous >3 years.

Comparative genome analysis, phylogenetic analysis using typical genes, and MALDI-TOF mass spectrometry analysis indicated that 2019-19 classified into the *H. haemolyticus* cluster rather than *H. influenzae*. Absence of IgA protease supported these results (*8*). In addition, this strain contained CRISPR sequences. Comparing genome sequences in the database, all *H. haemolyticus* contained CRISPR but *H. influenzae* sequences did not, which might support that this strain was *H. haemolyticus*. In contrast, 2019-19 shared biochemical features with *H. influenzae*. The biotype of 2019-19, biotype II, is the predominant type among *H. influenzae* and comparatively rare among *H. haemolyticus* (*17*,*26*,*27*), making it a notable feature of *H. haemolyticus* 2019-19. Previous studies reported that clinical isolates identified as *H. influenzae* occasionally included *H. haemolyticus* without hemolysis (*6*–*8*). The biochemical features of 2019-19 likely contributed to this misidentification. Genomic analysis showed 2019-19 contained a large inversion compared with other *H. haemolyticus* strains; however, the relationship of this inversion with biochemical features was not determined. Bacterial species defined by whole-genome sequence similarity have been reported to be ≈95%–96% ANIb (*28*) or 70% DDH (*29*). Although we tentatively identified 2019-19 as *H. haemolyticus*, these definitions and our ANIb and dDDH values suggest that this strain is a novel subspecies or species. The classification data for *Haemophilus* spp. are inadequate compared with those of other pathogens and species may need to be reclassified after additional genome and biochemical data are accumulated. Our findings can help improve the accuracy of classification and 2019-19 may be designated a novel subspecies or species in the future (*6*,*30*).

*H. haemolyticus* 2019-19 showed high-level quinolone-resistance and multiple amino acid substitutions in quinolone-targeting proteins, which are known to contribute to high-level quinolone resistance (*12*–*14*,*17*,*31*). In addition, quinolone-resistant *H. parainfluenzae* has been reported (*32*,*33*) in Taiwan and Europe and these isolates showed various amino acid substitutions in quinolone target genes, like those observed in 2019-19. Frequent use of quinolone can contribute to the emergence of resistant strains, and although this patient had not been exposed to any quinolones during the previous >3 years, she had frequently stayed in medical facilities and other antimicrobial agents had been used to treat her multiple coexisting diseases. Moreover, quinolones have been used for pediatric patients in Japan and the frequency of low-susceptibility strains of *H. influenzae* has been increasing (*9*,*10*,*34*). There may be selective pressure not only in hospitals but also in communities. In fact, 2019-19 contained a large inversion in the genome and partially differed from *H. haemolyticus*, suggesting substantial genetic recombination and rearrangement for this strain.

Among the study’s limitations, we analyzed only 1 high-level quinolone-resistant *H. haemolyticus* and its prevalence in both community and clinical settings remains unclear. In addition, there was no evidence about whether 2019-19 is a causative pathogen or commensal strain. However, the presence of high-level antimicrobial-resistant *Haemophilus* spp. in children should be noted, because even commensal bacteria can cause lethal infections in immunocompromised hosts.

The reasons why high-level quinolone-resistant *Haemophilus* spp. had not been isolated from children are unclear. A previous report suggested that quinolones have not been used to treat pediatric infections (*11*). In addition, because *H. influenzae* is a commensal nasopharyngeal bacteria for most children (*35*), quinolone-resistant strains may be outcompeted by other commensal bacteria. The adaptability of quinolone-resistant strains should be further analyzed.

In conclusion, our findings reveal the existence of high-level quinolone-resistant *Haemophilus* spp. strains in children. Horizontal gene transfer between *H. influenzae* and *H. haemolyticus* has been observed (*36*,*37*), and high-level quinolone-resistant *H. influenzae* may also emerge. Therefore, the presence of high-level resistance strains should be considered when quinolones are used to treat children. 

Appendix Additional information on quinolone-resistant *Haemophilus haemolyticus* in a pediatric patient, Japan.
