# Supplementary material for: High-Level Quinolone-Resistant Haemophilus haemolyticus in Pediatric Patient with No History of Quinolone Exposure
Source: Emerg Infect Dis. 2022 Jan;28(1):104–10. doi: 10.3201/eid2801.210248 (PMC8714209; doi:10.3201/eid2801.210248)
Supplement: Appendix — Additional information on quinolone-resistant Haemophilus haemolyticus in a pediatric patient, Japan. [file 21-0248-Techapp-s1.pdf]

Article DOI: https://doi.org/10.3201/eid2801.210248

Appendix

High-Level Quinolone-Resistant *Haemophilus haemolyticus* in a Pediatric Patient with No History of Quinolone Exposure

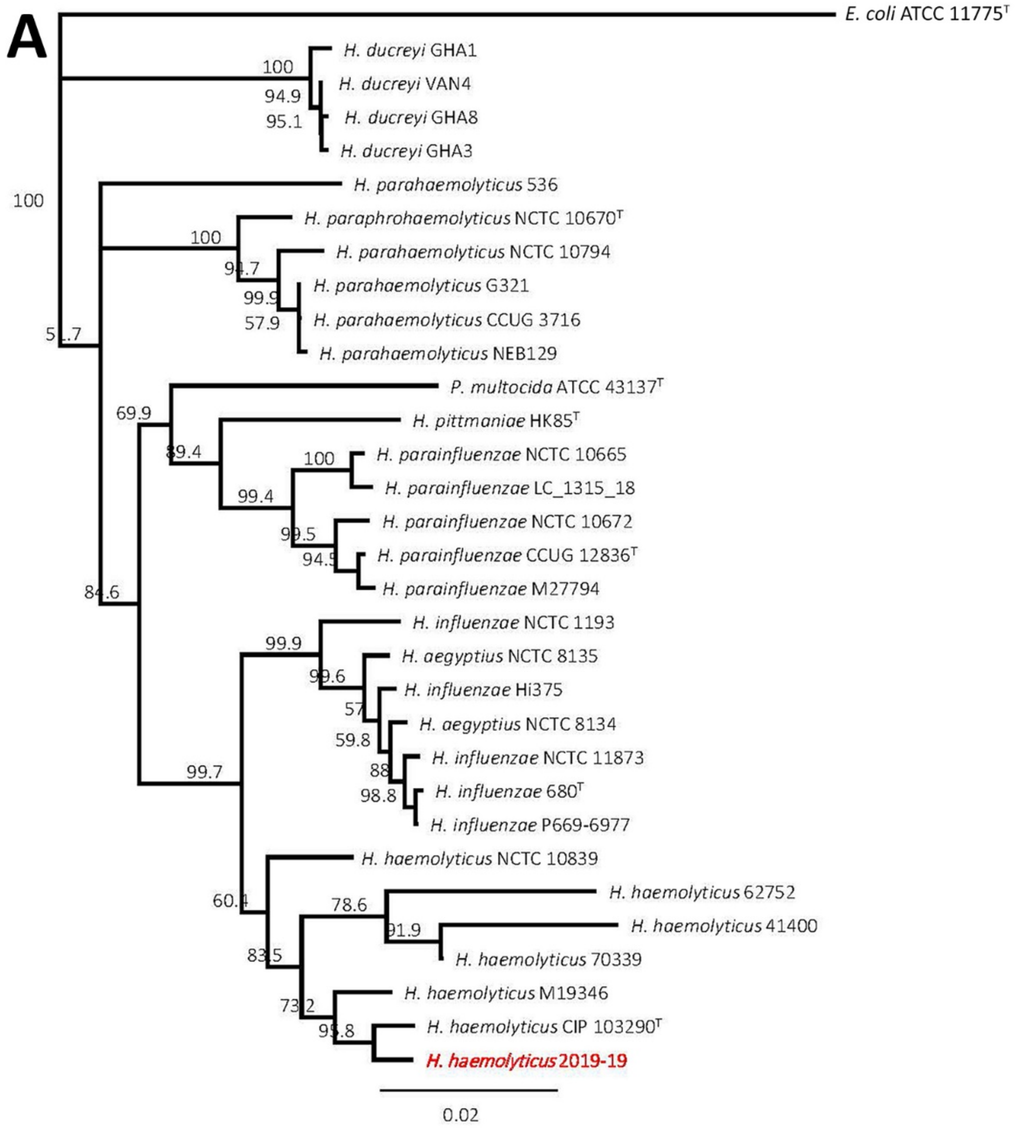

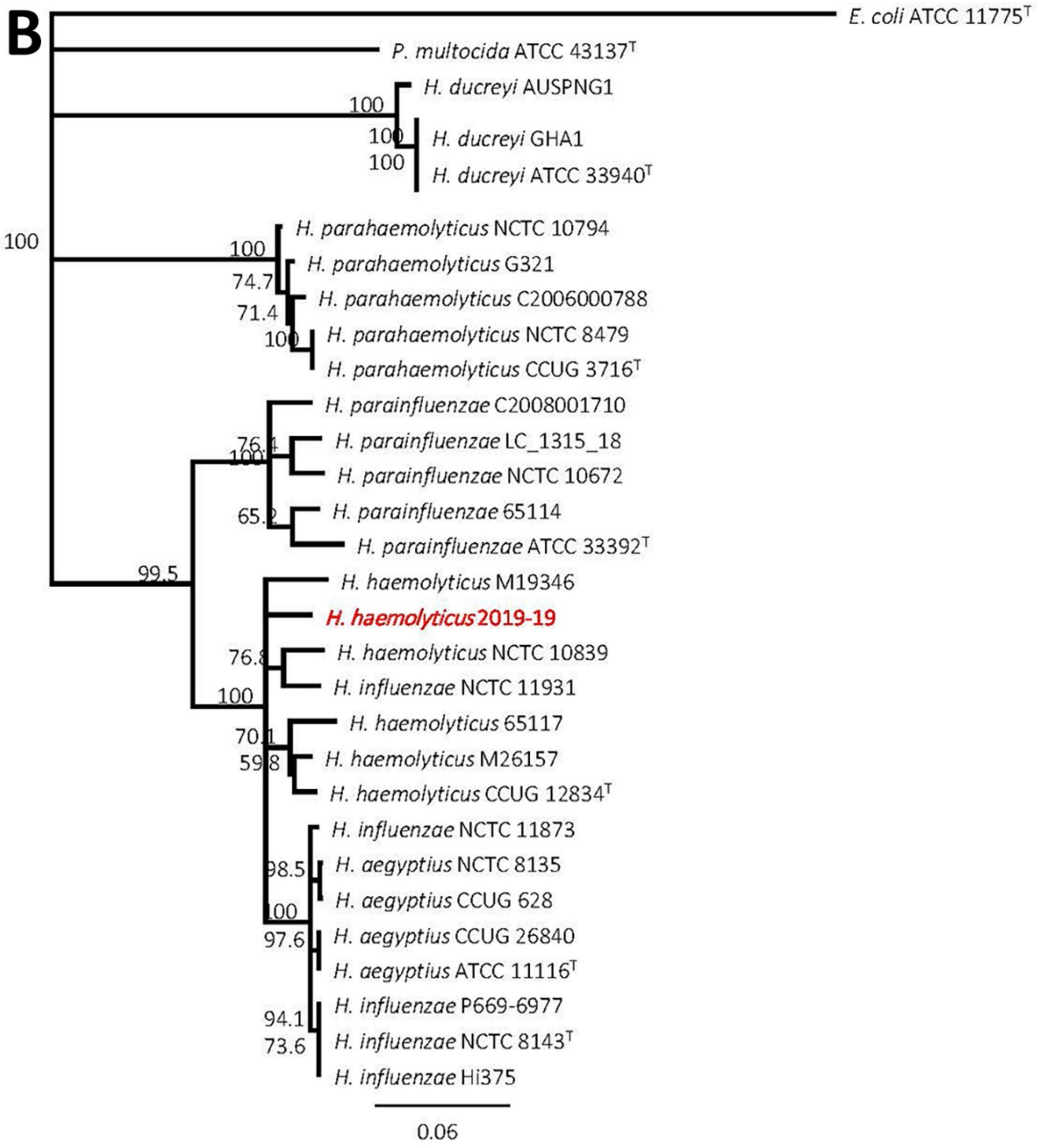
B
E. coli ATCC 11775T
P. multocida ATCC 43137T
H. ducreyi AUSPNG1
H. ducreyi GHA1
H. ducreyi ATCC 33940T
H. parahaemolyticus NCTC 10794
H. parahaemolyticus G321
H. parahaemolyticus C2006000788
H. parahaemolyticus NCTC 8479
H. parahaemolyticus CCUG 3716T
H. parainfluenzae C2008001710
H. parainfluenzae LC_1315_18
H. parainfluenzae NCTC 10672
H. parainfluenzae 65114
H. parainfluenzae ATCC 33392T
H. haemolyticus M19346
H. haemolyticus 2019-19
H. haemolyticus NCTC 10839
H. influenzae NCTC 11931
H. haemolyticus 65117
H. haemolyticus M26157
H. haemolyticus CCUG 12834T
H. influenzae NCTC 11873
H. aegyptius NCTC 8135
H. aegyptius CCUG 628
H. aegyptius CCUG 26840
H. aegyptius ATCC 11116T
H. influenzae P669-6977
H. influenzae NCTC 8143T
H. influenzae Hi375
100
100
100
100
100
74.7
71.4
100
76.4
100
65.2
99.5
76.8
100
70.1
59.8
98.5
100
97.6
94.1
73.6
0.06

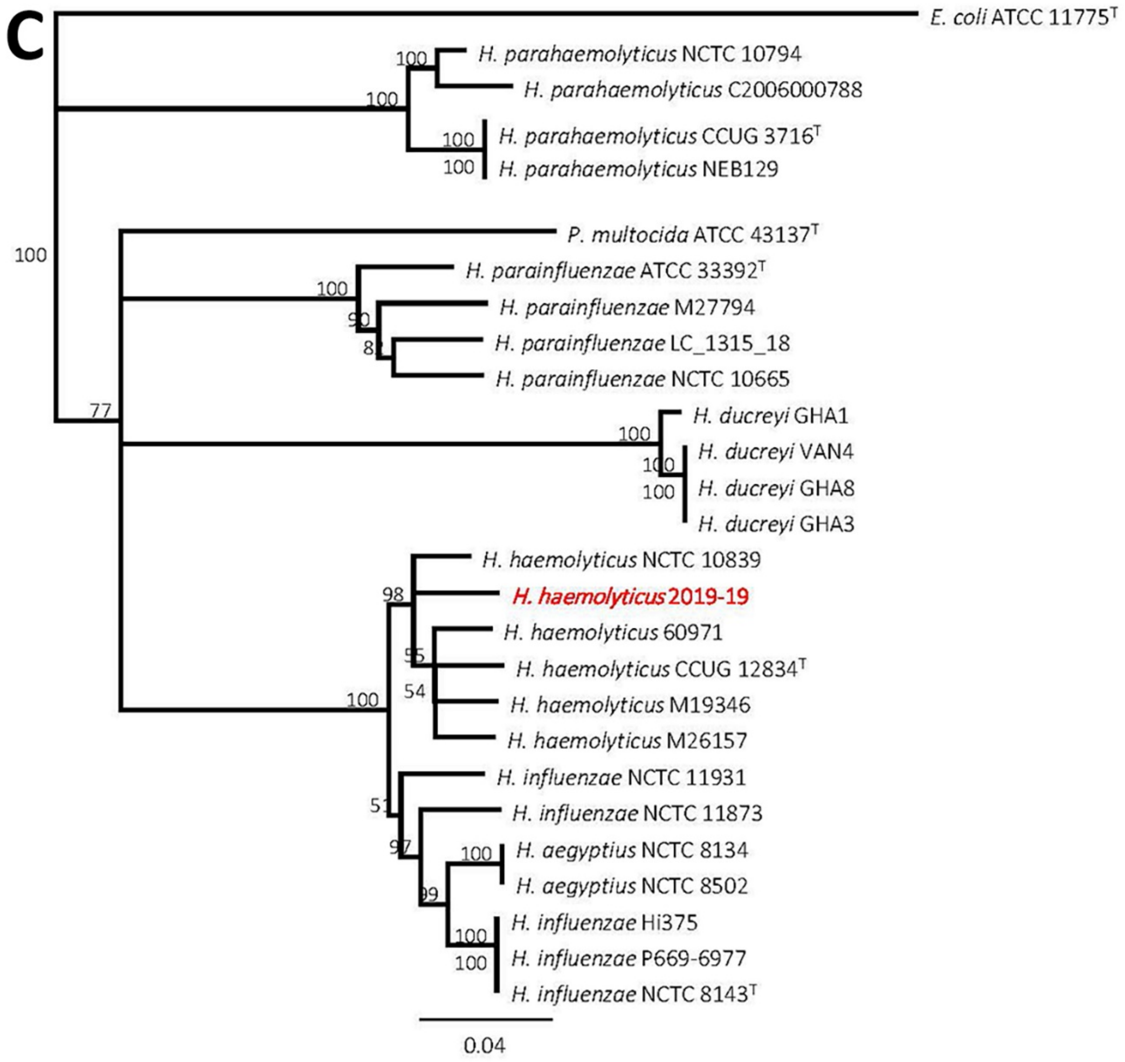
C
E. coli ATCC 11775T
H. parahaemolyticus NCTC 10794
H. parahaemolyticus C2006000788
H. parahaemolyticus CCUG 3716T
H. parahaemolyticus NEB129
P. multocida ATCC 43137T
H. parainfluenzae ATCC 33392T
H. parainfluenzae M27794
H. parainfluenzae LC_1315_18
H. parainfluenzae NCTC 10665
H. ducreyi GHA1
H. ducreyi VAN4
H. ducreyi GHA8
H. ducreyi GHA3
H. haemolyticus NCTC 10839
H. haemolyticus 2019-19
H. haemolyticus 60971
H. haemolyticus CCUG 12834T
H. haemolyticus M19346
H. haemolyticus M26157
H. influenzae NCTC 11931
H. influenzae NCTC 11873
H. aegyptius NCTC 8134
H. aegyptius NCTC 8502
H. influenzae Hi375
H. influenzae P669-6977
H. influenzae NCTC 8143T
0.04

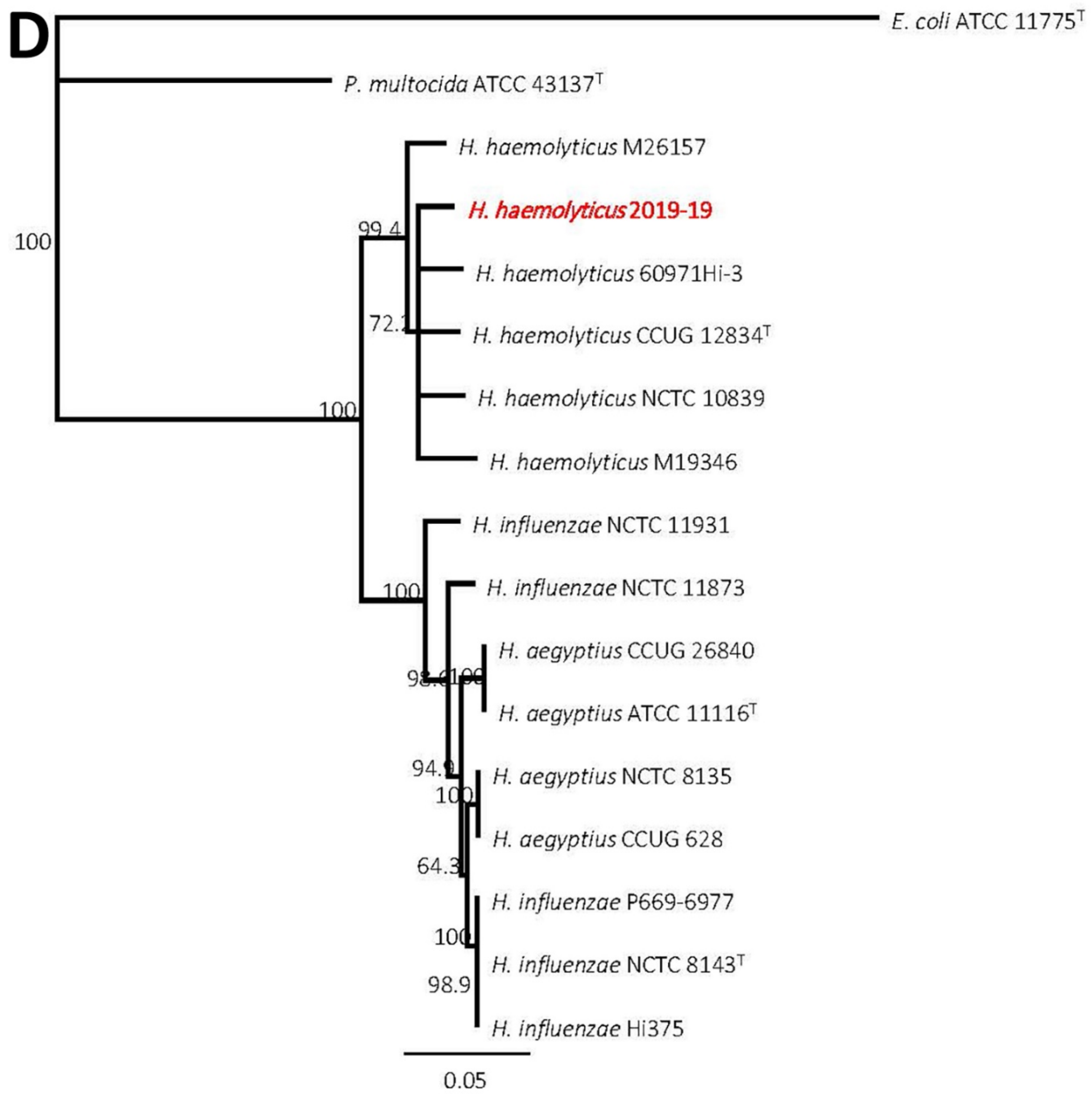
D
E. coli ATCC 11775T
P. multocida ATCC 43137T
H. haemolyticus M26157
H. haemolyticus 2019-19
H. haemolyticus 60971Hi-3
H. haemolyticus CCUG 12834T
H. haemolyticus NCTC 10839
H. haemolyticus M19346
H. influenzae NCTC 11931
H. influenzae NCTC 11873
H. aegyptius CCUG 26840
H. aegyptius ATCC 11116T
H. aegyptius NCTC 8135
H. aegyptius CCUG 628
H. influenzae P669-6977
H. influenzae NCTC 8143T
H. influenzae Hi375
100
99.4
72.1
100
100
94.9
100
64.3
100
98.9
0.05

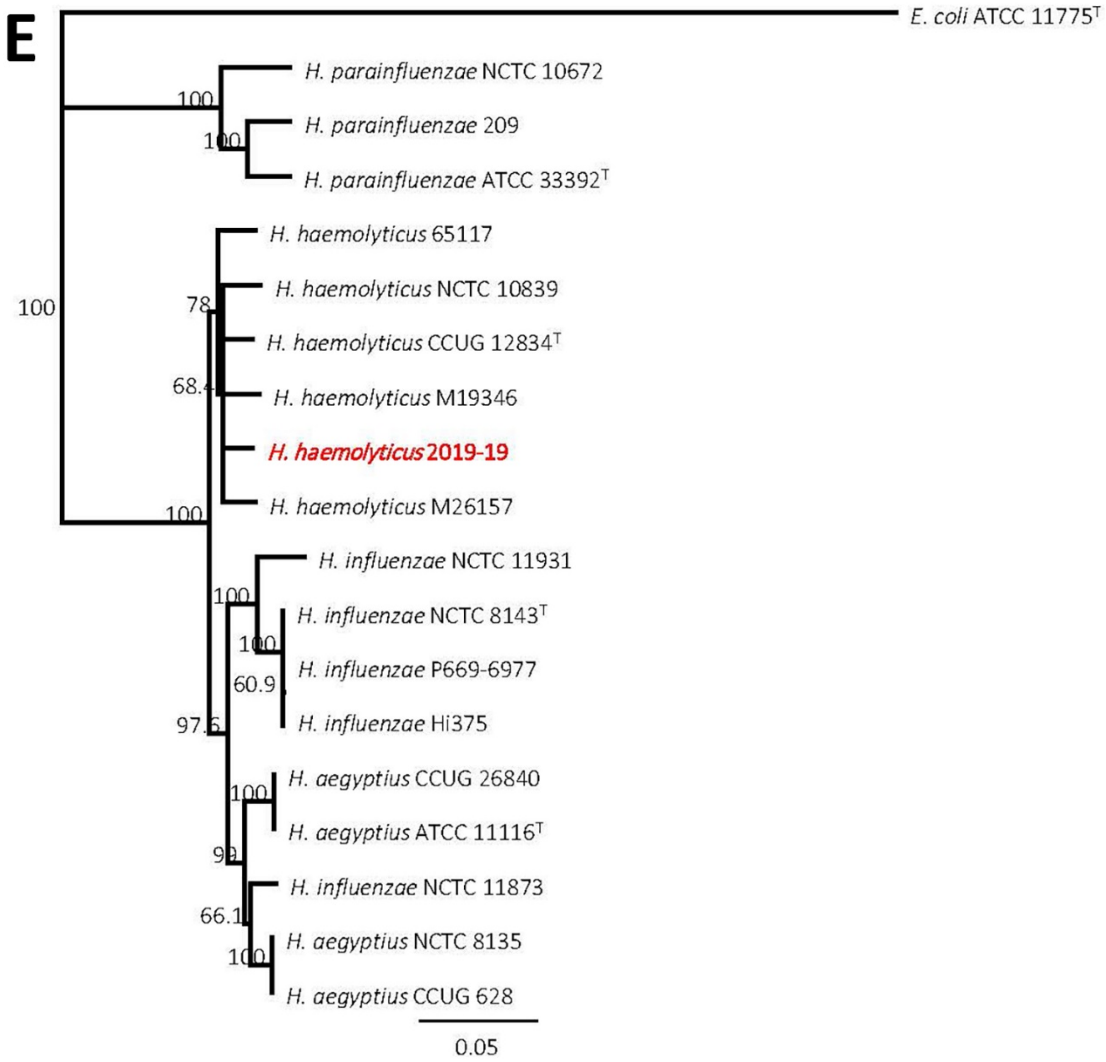
E
E. coli ATCC 11775T
H. parainfluenzae NCTC 10672
H. parainfluenzae 209
H. parainfluenzae ATCC 33392T
H. haemolyticus 65117
H. haemolyticus NCTC 10839
H. haemolyticus CCUG 12834T
H. haemolyticus M19346
H. haemolyticus 2019-19
H. haemolyticus M26157
H. influenzae NCTC 11931
H. influenzae NCTC 8143T
H. influenzae P669-6977
H. influenzae Hi375
H. aegyptius CCUG 26840
H. aegyptius ATCC 11116T
H. influenzae NCTC 11873
H. aegyptius NCTC 8135
H. aegyptius CCUG 628
100
100
100
78
68.4
100
100
100
60.9
97.6
100
99
66.1
100
0.05

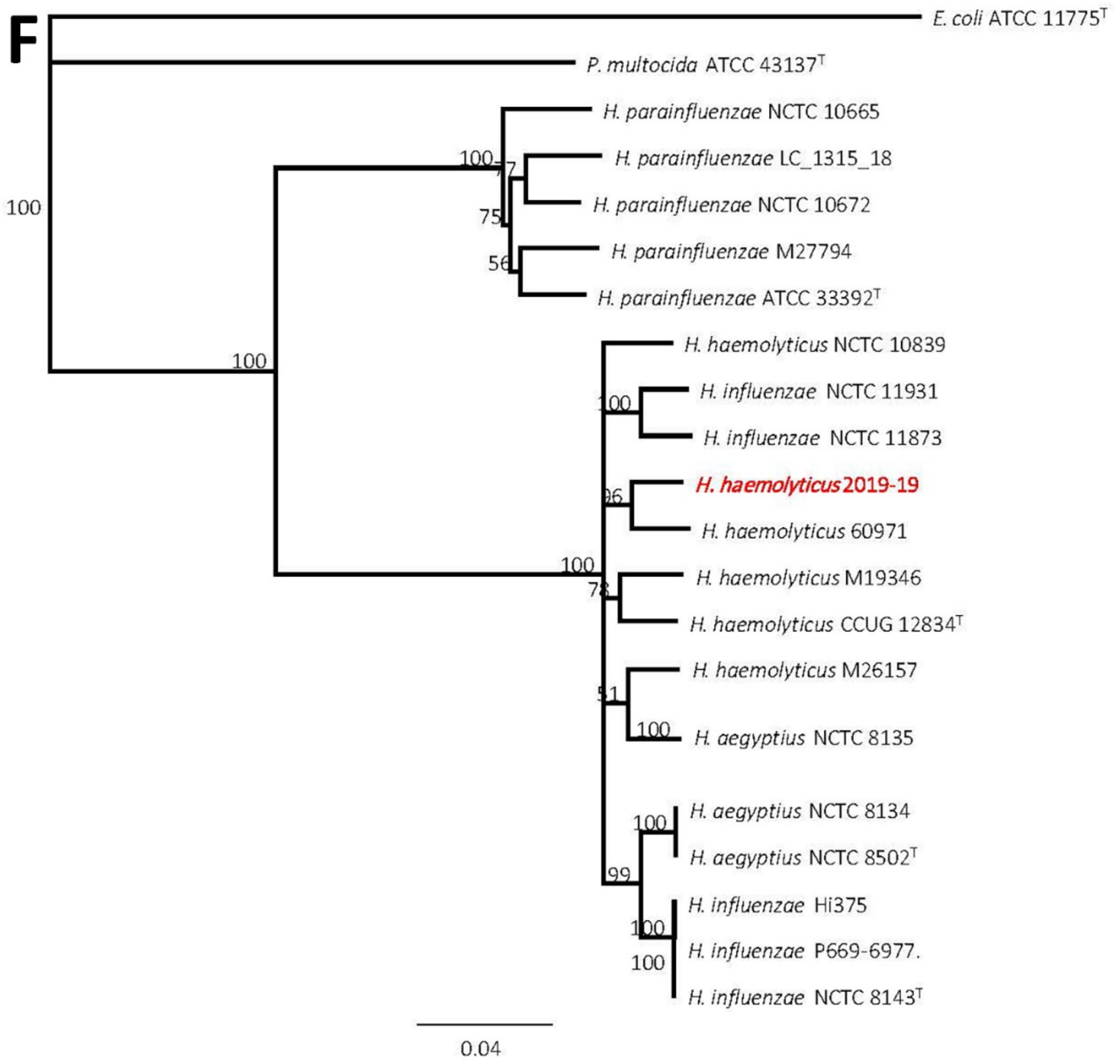
F
E. coli ATCC 11775T
P. multocida ATCC 43137T
H. parainfluenzae NCTC 10665
H. parainfluenzae LC_1315_18
H. parainfluenzae NCTC 10672
H. parainfluenzae M27794
H. parainfluenzae ATCC 33392T
H. haemolyticus NCTC 10839
H. influenzae NCTC 11931
H. influenzae NCTC 11873
H. haemolyticus 2019-19
H. haemolyticus 60971
H. haemolyticus M19346
H. haemolyticus CCUG 12834T
H. haemolyticus M26157
H. aegyptius NCTC 8135
H. aegyptius NCTC 8134
H. aegyptius NCTC 8502T
H. influenzae Hi375
H. influenzae P669-6977.
H. influenzae NCTC 8143T
0.04

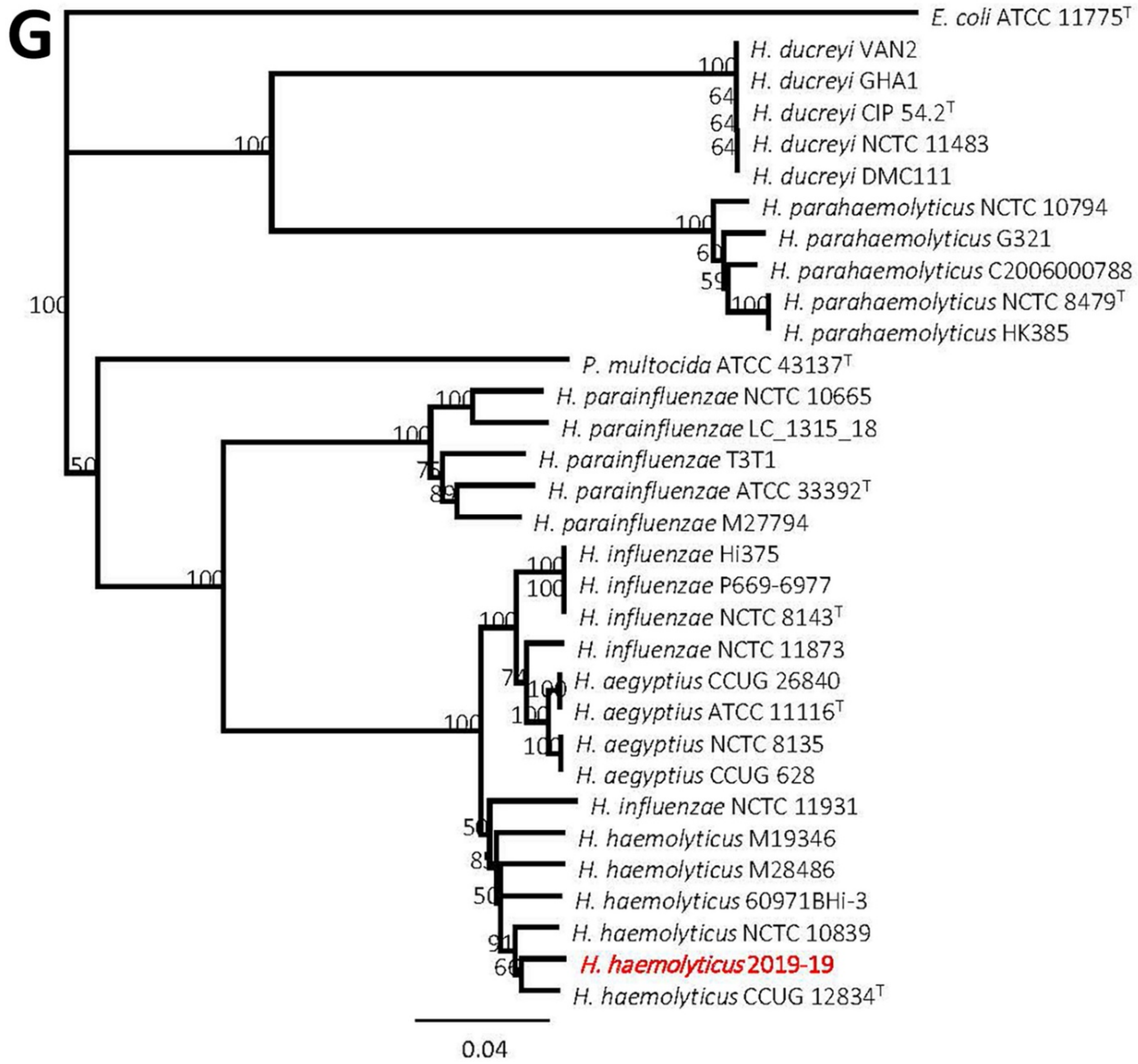
G
E. coli ATCC 11775T
H. ducreyi VAN2
H. ducreyi GHA1
H. ducreyi CIP 54.2T
H. ducreyi NCTC 11483
H. ducreyi DMC111
H. parahaemolyticus NCTC 10794
H. parahaemolyticus G321
H. parahaemolyticus C2006000788
H. parahaemolyticus NCTC 8479T
H. parahaemolyticus HK385
P. multocida ATCC 43137T
H. parainfluenzae NCTC 10665
H. parainfluenzae LC_1315_18
H. parainfluenzae T3T1
H. parainfluenzae ATCC 33392T
H. parainfluenzae M27794
H. influenzae Hi375
H. influenzae P669-6977
H. influenzae NCTC 8143T
H. influenzae NCTC 11873
H. aegyptius CCUG 26840
H. aegyptius ATCC 11116T
H. aegyptius NCTC 8135
H. aegyptius CCUG 628
H. influenzae NCTC 11931
H. haemolyticus M19346
H. haemolyticus M28486
H. haemolyticus 60971BHi-3
H. haemolyticus NCTC 10839
H. haemolyticus 2019-19
H. haemolyticus CCUG 12834T
0.04

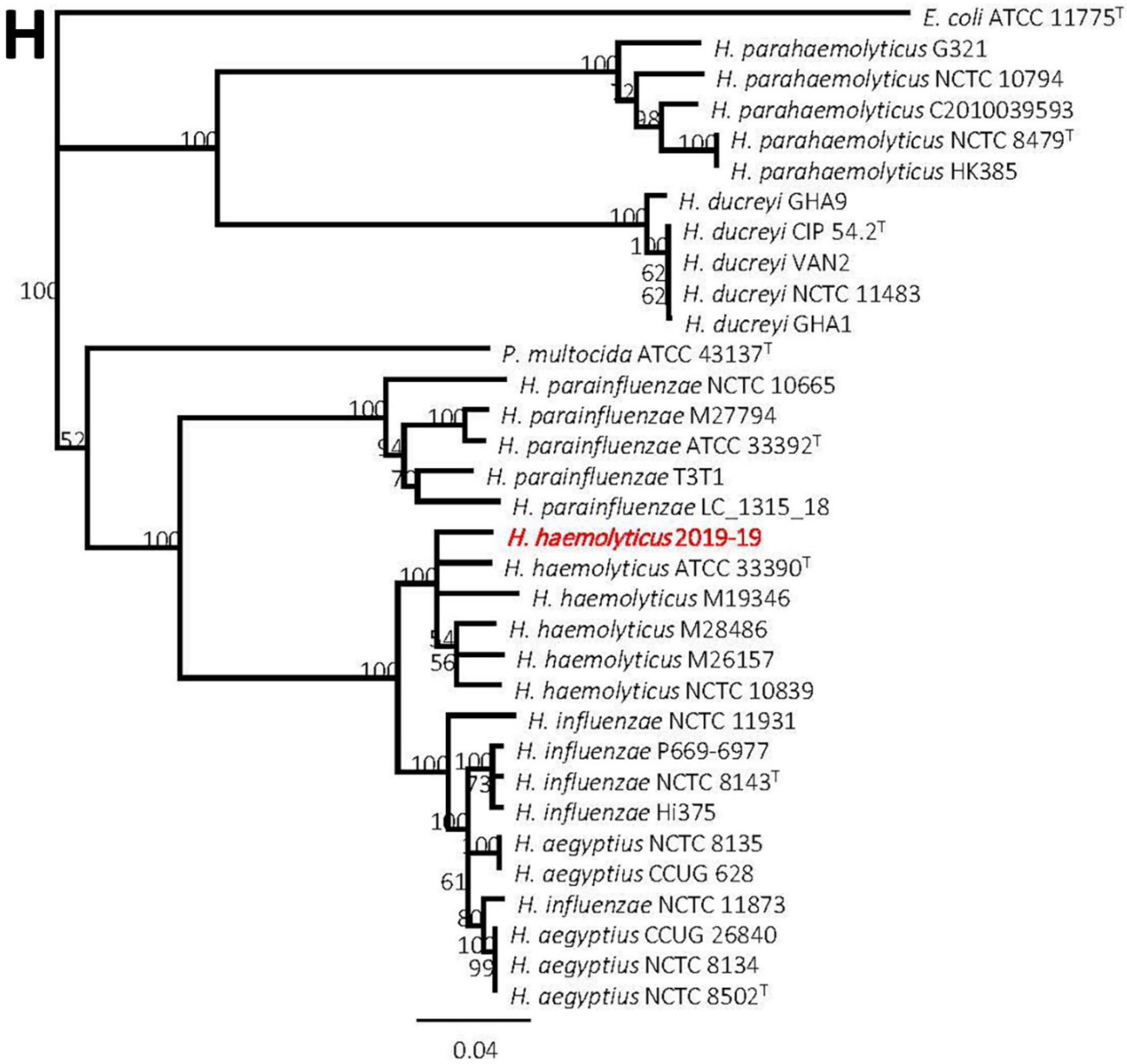
H
E. coli ATCC 11775T
H. parahaemolyticus G321
H. parahaemolyticus NCTC 10794
H. parahaemolyticus C2010039593
H. parahaemolyticus NCTC 8479T
H. parahaemolyticus HK385
H. ducreyi GHA9
H. ducreyi CIP 54.2T
H. ducreyi VAN2
H. ducreyi NCTC 11483
H. ducreyi GHA1
P. multocida ATCC 43137T
H. parainfluenzae NCTC 10665
H. parainfluenzae M27794
H. parainfluenzae ATCC 33392T
H. parainfluenzae T3T1
H. parainfluenzae LC_1315_18
H. haemolyticus 2019-19
H. haemolyticus ATCC 33390T
H. haemolyticus M19346
H. haemolyticus M28486
H. haemolyticus M26157
H. haemolyticus NCTC 10839
H. influenzae NCTC 11931
H. influenzae P669-6977
H. influenzae NCTC 8143T
H. influenzae Hi375
H. aegyptius NCTC 8135
H. aegyptius CCUG 628
H. influenzae NCTC 11873
H. aegyptius CCUG 26840
H. aegyptius NCTC 8134
H. aegyptius NCTC 8502T
0.04

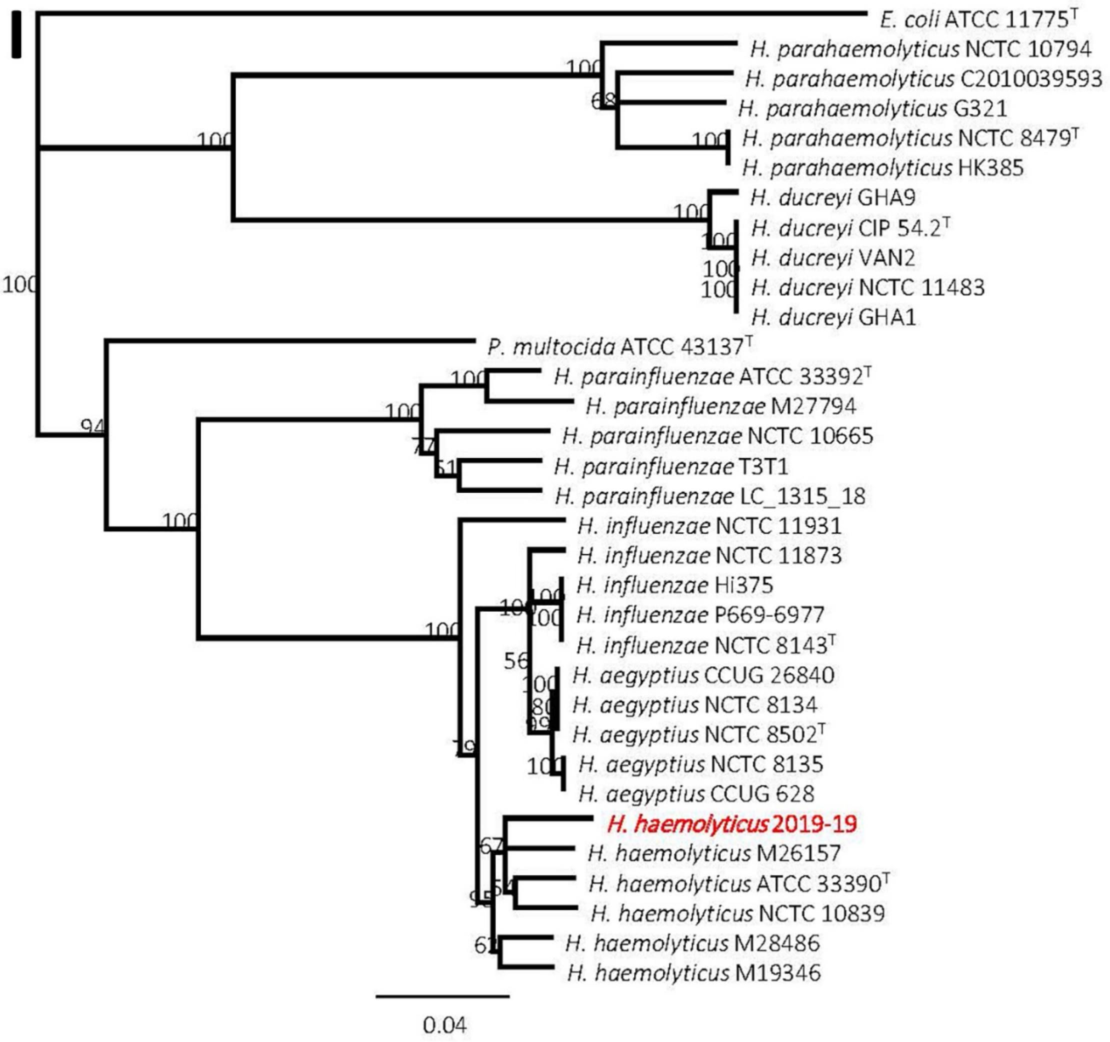
E. coli ATCC 11775T
H. parahaemolyticus NCTC 10794
H. parahaemolyticus C2010039593
H. parahaemolyticus G321
H. parahaemolyticus NCTC 8479T
H. parahaemolyticus HK385
H. ducreyi GHA9
H. ducreyi CIP 54.2T
H. ducreyi VAN2
H. ducreyi NCTC 11483
H. ducreyi GHA1
P. multocida ATCC 43137T
H. parainfluenzae ATCC 33392T
H. parainfluenzae M27794
H. parainfluenzae NCTC 10665
H. parainfluenzae T3T1
H. parainfluenzae LC_1315_18
H. influenzae NCTC 11931
H. influenzae NCTC 11873
H. influenzae Hi375
H. influenzae P669-6977
H. influenzae NCTC 8143T
H. aegyptius CCUG 26840
H. aegyptius NCTC 8134
H. aegyptius NCTC 8502T
H. aegyptius NCTC 8135
H. aegyptius CCUG 628
H. haemolyticus 2019-19
H. haemolyticus M26157
H. haemolyticus ATCC 33390T
H. haemolyticus NCTC 10839
H. haemolyticus M28486
H. haemolyticus M19346
0.04

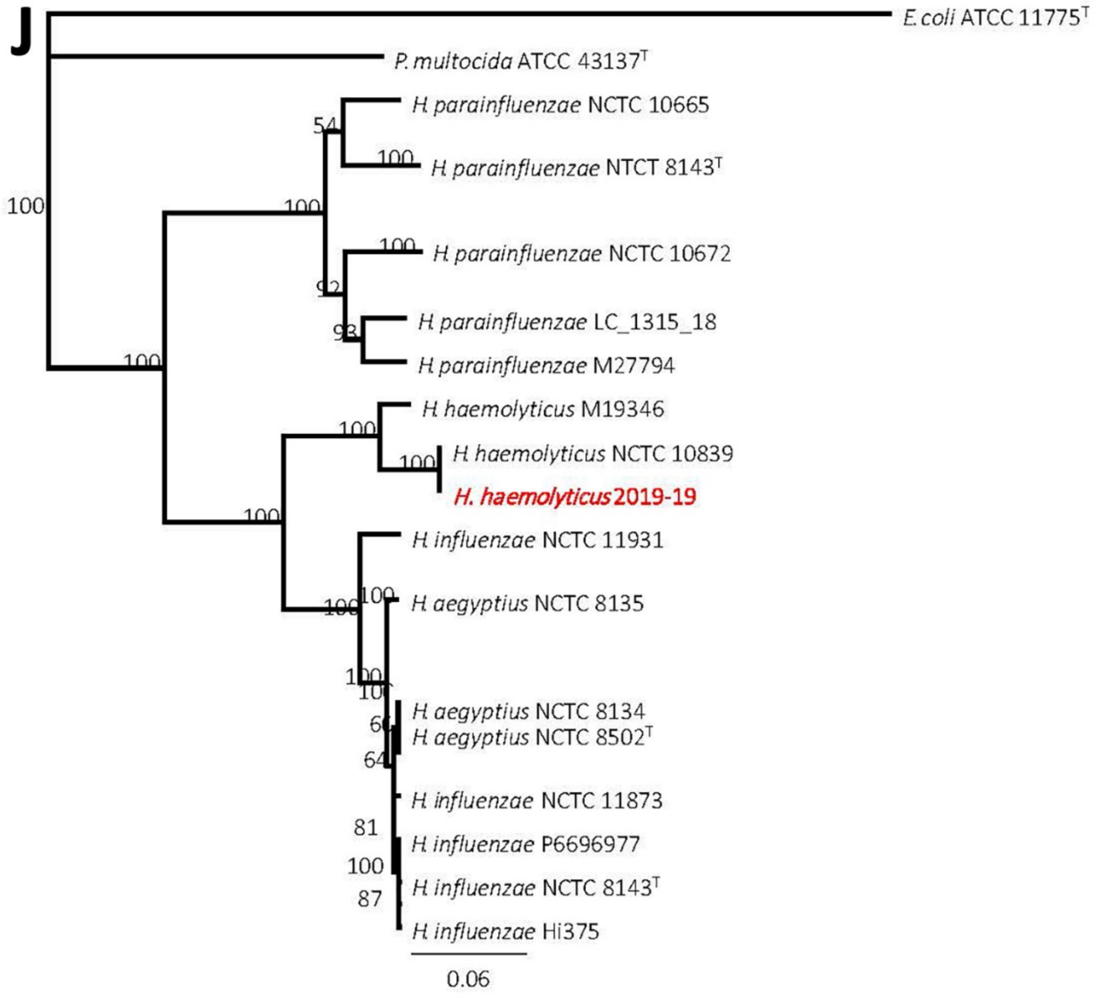

Appendix Figure 1. Neighbor-joining dendrograms based on nucleotide sequences. The dendrograms were drawn based on the nucleotide sequence of typical genes. They were drawn using the Clustal Omega program and neighbor-joining method using Geneious prime 2019 (Biomatters, https://www.geneious.com). *Escherichia coli* ATCC 11775^T was considered an outgroup. Panels A) 16S rDNA nucleotide sequences, B) *adk*, C) *pgi*, D) *recA*, E) *infB*, F) *gyrA*, G) *gyrB*, H) *parC*, I) *parE*, and J) *ftsI*. *H.*, *Haemophilus*

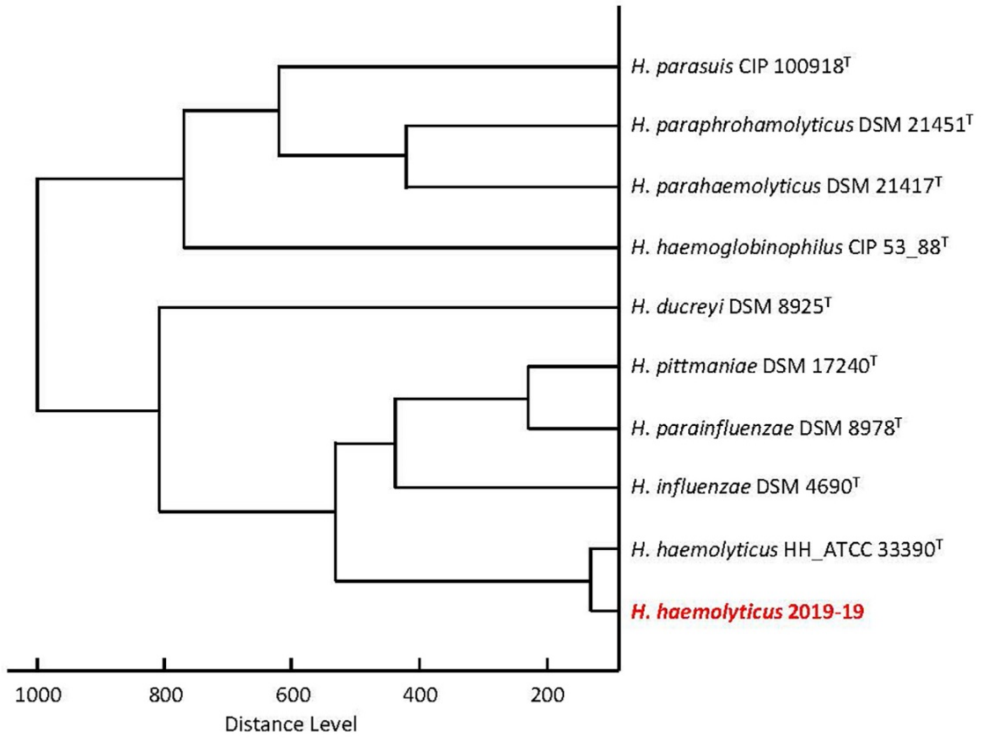

Appendix Figure 2. Main spectra library dendrogram of MALDI-TOF mass spectral profiles. The dendrogram was generated using MBT Compass Explorer 4.1.60 software (Bruker, https://www.bruker.com) on the basis of the MALDI-TOF mass spectral profiles of 10 *Haemophilus* spp. *H.*, *Haemophilus*

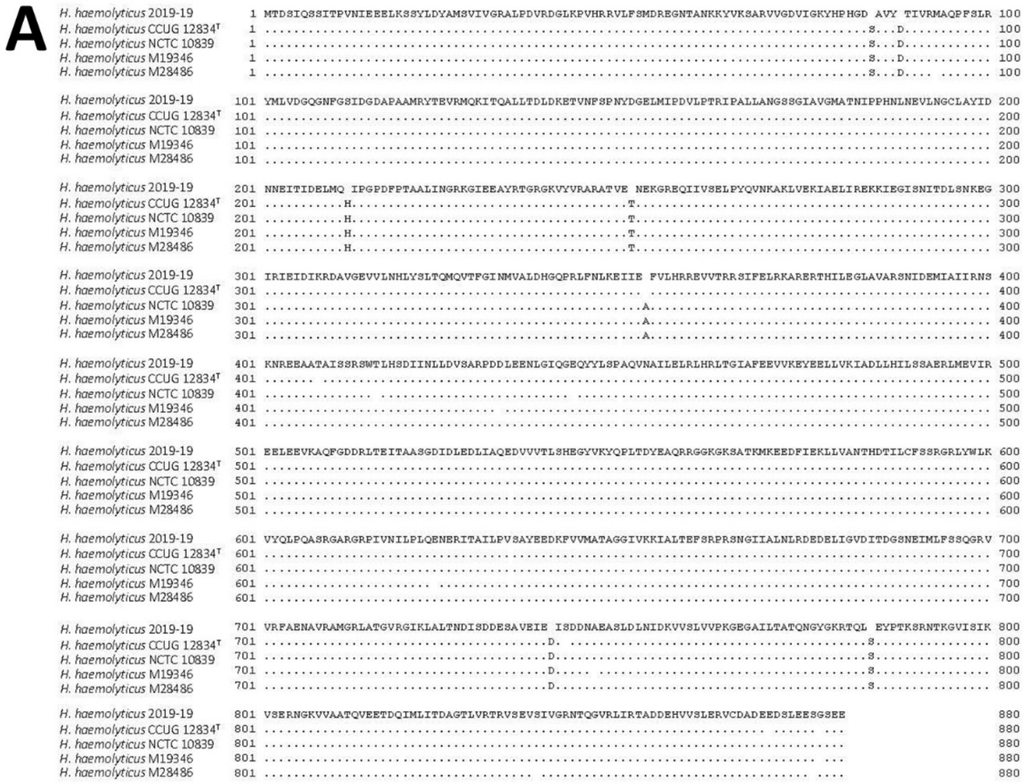
A
H. haemolyticus 2019-19
H. haemolyticus CCUG 12834T
H. haemolyticus NCTC 10839
H. haemolyticus M19346
H. haemolyticus M28486

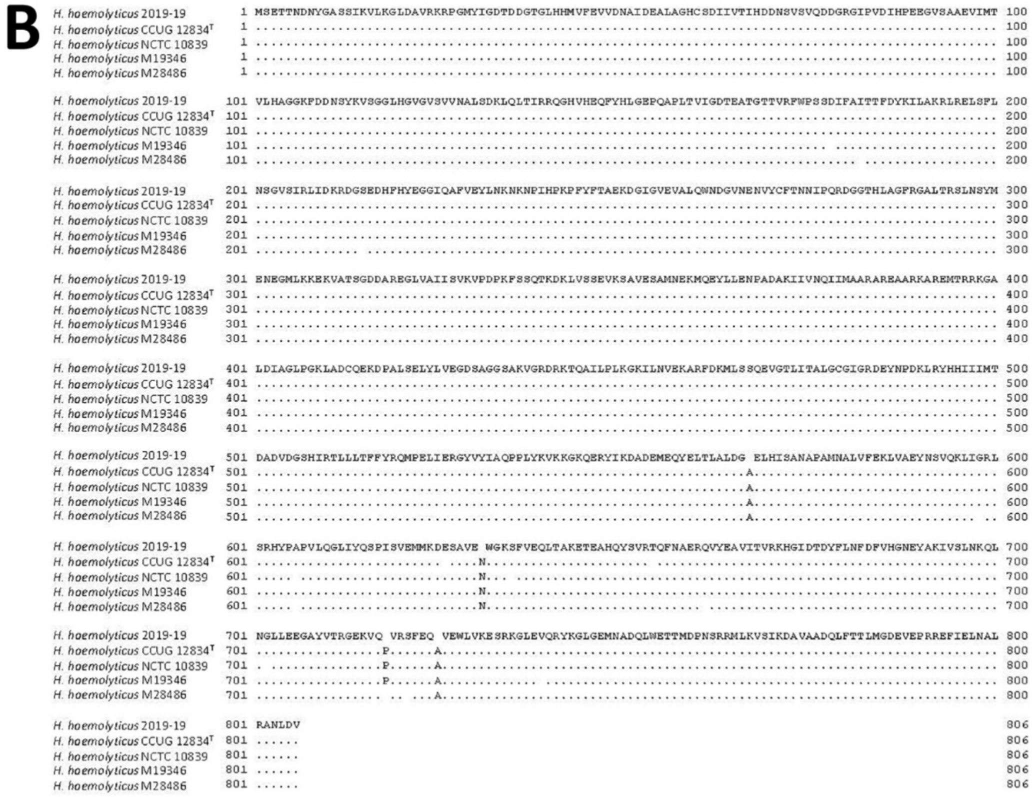
B
H. haemolyticus 2019-19
H. haemolyticus CCUG 12834T
H. haemolyticus NCTC 10839
H. haemolyticus M19346
H. haemolyticus M28486

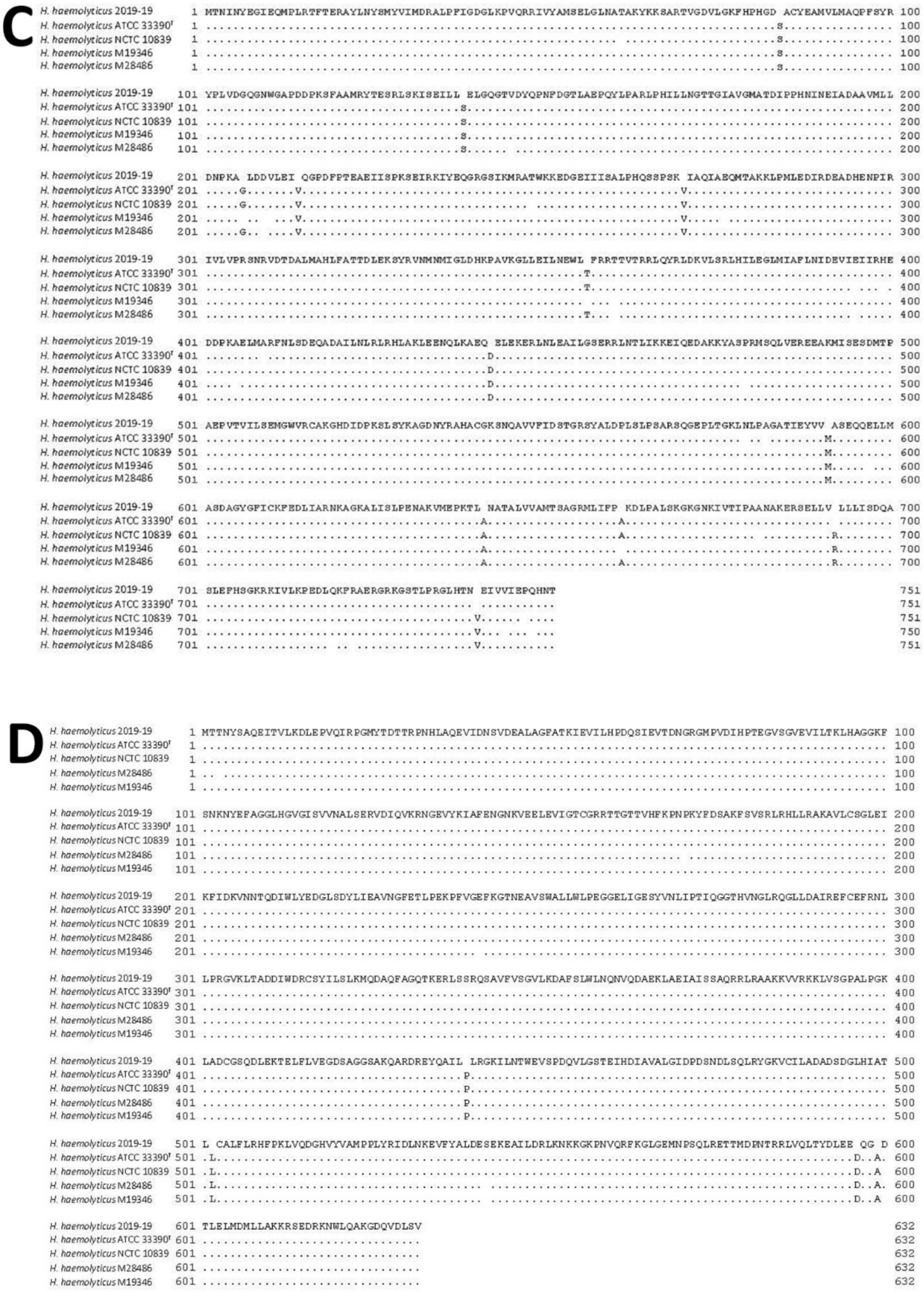

Appendix Figure 3. Amino acid alignments of quinolone target proteins. These alignments were created using Genetyx version 10 (https://www.genetyx.co.jp). The amino acid substitutions are shown as single character codes. These data are the basis for Table 3. Panels A) *gyrA*, B) *gyrB*, C) *parC*, and D) *parE*. *H.*, *Haemophilus*
